# Prevention of anastomotic fistula formation after low-position Dixon Operation 

**DOI:** 10.12669/pjms.305.4453

**Published:** 2014

**Authors:** Feng Gao, Ming Xu, Feng Song, Xin Zhang, Yong Zhao

**Affiliations:** 1 Feng Gao, Department of General Surgery in VIP Ward, Lanzhou Command General Hospital, Lanzhou 730050, China.; 2 Ming Xu, Department of General Surgery in VIP Ward, Lanzhou Command General Hospital, Lanzhou 730050, China.; 3 Feng Song, Department of General Surgery in VIP Ward, Lanzhou Command General Hospital, Lanzhou 730050, China.; 4 Xin Zhang, Department of General Surgery in VIP Ward, Lanzhou Command General Hospital, Lanzhou 730050, China.; 5 Yong Zhao, Department of General Surgery in VIP Ward, Lanzhou Command General Hospital, Lanzhou 730050, China.

**Keywords:** Anastomotic Fistula, Mayo Clinic Operation, Colorectal Cancer, Skill

## Abstract

***Objective:*** This study aimed to investigate the main points of preventing anastomotic fistula formation after low-position Dixon operation.

***Methods:*** From September 2004 to October 2007, our department continuously conducted 146 cases of low-position Dixon operations. The operation mode involved transabdominal radical resection based on total mesorectal excision for all cases. Except for tumor infiltration, one side of the pelvic vegetative nerve was maintained and ligations were conducted at the superior rectal artery root. Mesorectum at the anastomosis site was removed up to the tunica muscularis recti. The anastomotic stoma blood supply was good and had no tension. An anal tube was inserted when the anastomotic stoma was within 3 cm away from the anal margin. For all cases, a presacral drainage tube was placed via the perineal position.

***Results: ***For all 146 cases, no anastomotic leakage occurred and the post-operative complications included two cases of anastomotic bleeding, three cases of anastomotic stenoses, 48 cases of increased defecation (4-6 times of defecation daily), 34 cases of anal irritation symptoms, and 6 cases of poor loose stool control capacities.

***Conclusion:*** Ensuring enough blood supply for the anastomotic bowel on the two sides, eliminating tension and accurate anastomosis at the anastomosis site could be effective measures to prevent anastomotic fistula in the low position anus preserving surgery of colorectal cancer.

## INTRODUCTION

Anastomotic fistula formation is one of the most serious complications of low rectal cancer after Dixon operation, and some patients lose their lives. Although doctors performing colorectal surgery have used multiple measures to prevent anastomotic fistula formation for many years, the results are still unsatisfactory. The incidence of anastomotic leakage after Dixon operation is reportedly about 2.0%-22.0%.^1^^,^^2^ The incidence of anastomotic fistula has increased in recent years because of the application of total mesorectal excision (TME).^3^ More than six times increase in anastomotic fistula were reported by Law and Chu after TME operation compared with conventional operation.^4^

## METHODS

From September 2004 to October 2007, our department continuously performed 146 cases of low-position Dixon operations. The patients included 99 males and 47 females. Their ages ranged from 26 years to 85 years. The disease distribution was as follows: 4 cases of malignant changes of villous adenoma, 2 cases of villous adenomas, 2 cases of wide base tubular adenomas, 1 case of rectal cancer, 2 cases of rectal stromal tumors, 18 cases of well-differentiated adenocarcinomas, 89 cases of moderately differentiated adenocarcinomas, and 28 cases of poorly differentiated adenocarcinoma. The different tumor positions were as follows: 29 cases were over pelvic floor peritoneum reflection, 62 cases were horizontal to the peritoneum reflection, and 55 cases were below peritoneum reflection. Among the 146 cases, 27, 64, 52, 3 cases were in TNM I, II, III and IV stages, respectively. This study was conducted in accordance with the declaration of Helsinki after approval from the Ethics Committee of Lanzhou Command General Hospital. Written informed consent was obtained from all participants.

***Operation procedure:*** Transabdominal radical resection was performed based on TME for all cases. The method of maintaining pelvic vegetative nerves was used for most cases to at least maintain one side of the nerve, except for patients with tumors severely infiltrating outward. Double ligation was conducted at the superior rectal artery root, and the root of the inferior mesenteric artery was not ligated regularly. Double anastomat and single anastomat were applied in the anus-preserving operation to the 126 and 20 cases, respectively. Anastomosis was conducted with the single anastomat; the rectal stump was intermittently sutured and closed firstly, and the remaining steps were same as described for the double anastomat. If the closed rectal stump bled when the double anastomat was used, it was intermittently sutured in curve eight again. Mesorectum at the anastomosis site was removed completely up to the tunica muscularis recti. However, avoiding damage to the intestinal wall was necessary. After low-position Dixon operation was conducted, the transverse colostomy was additionally performed for one case of patient receiving preoperative radiotherapy. For the other 145 cases, prophylactic colostomy was not conducted. Drainage tube was implanted into the rectum through the anus, according to the author’s personal experience, to prevent the formation of high pressure in the proximal intestinal lumen side when the bowel enterocinesia function recovered. A presacral drainage tube was placed via the perineal position for all cases. The presacral drainage tube was removed at 4-5 day post-operation or defecation via the anus. In addition, as the pelvic peritoneum was closed, the anastomotic stoma was placed outside of the peritoneum.

***Adjunctive therapy: ***Preoperative fluorouracil (5-Fu) retention enema (50-100 ml inorganic serum plus 250 mg 5-Fu, retention enema, quaque nocte) was performed for the 128 cases out of the 146 of patients until the operation day. Peripheral venous adjuvant chemotherapy was conducted for 36 cases (5% glucose injection 250 ml plus calcium folinate 300 mg, intravenous drip, d1-5; 0.9% sodium chloride injection 500 ml plus 5-Fu 500-750 mg, intravenous drip, d1-5), and the operation was conducted at 4-7 dayafter the completion of chemotherapy. Nineteen patients received the post-operative pelvic adjuvant radiotherapy. In addition, one case of patient received the preoperative radiotherapy, and the operation was conducted at 50 day after the completion of radiotherapy.

## RESULTS

The diameters of all tubular anastomat were as follows: 33, 32, 31, and 29 mm. The diameter of the anastomat used was 29 mm because the anastomotic positions of the nine cases were too low; placing an anastomat with a diameter over 31 mm into the anal tube was difficult. Anastomotic leakage based on clinical judgment was absent in all the 146 operations. Post-operative complications included 2 cases of anastomotic bleeding (double anastomat anastomosis, both were healed by conservative treatment), 3 cases of anastomotic stenoses (2 cases used double anastomat and 1 case used single anastomat; both were improved by scratching the anus), 48 cases of increased defecation (4-6 times of defecation daily), 34 cases of anal irritation symptoms and 6 cases of poor loose stool control capacities (cases were healed after enema treatment). The minimum and maximum distances of the inferior tumor margin away from the incisal margin were 1 cm and 4 cm, respectively, in case of no fixation. After fixation, the minimum and maximum lengths were 0.5 cm and 3 cm, respectively. From post-operative follow-up to date, only one case presented with local recurrence at 1.5 years post-operation, and Miles operation was conducted.

## DISCUSSION

Anastomotic fistula is the most distressing local complication after Dixon operation. If anastomotic fistula occurs, it can directly cause anus-preserving operation failure, aggravate patient pains, prolong hospitalization time, increase medical expenses, and reduce life quality.^5^ Although a number of conservative methods of treating anastomotic fistula are available, its cure rate is only about 50%, and about 50% of the patients require surgical treatment. Therefore, prevention against anastomotic fistula after Dixon operation is very important.^6^^,^^7^ Majority of scholars have studied the influences of single factors such as anastomotic tension, preventive enterostomy, and transfusion on anastomotic leakage.^3^^,^^8^^,^^9^ Based on our experience, among the 146 cases of low-position Dixon operations conducted by our department, anastomotic fistula did not occur. Comprehensively considering the patient's physical condition, operative procedure details, is necessary for the prevention of anastomotic leakage occurrence.

Ensuring blood supply to the anastomosis site is the key to prevent anastomotic fistula. The selection of mode of operation in low-position anus preservation depends on the specific situations of the patients. For patients with rectal cancer, adequate freeing of the flexura coli sinistra and evaluating the anatomy during operation on a TME level is necessary to highlight. Subsequently, the mode of operation of the anus preservation is selected according to the specific situations of the patients. Although some scholars assume that the incidence of anastomotic leakage after TME operation will be increased, we regard that this possibility is smaller. The blood supply at the two sides of the anastomotic stoma is the key to prevent anastomotic fistula. Some scholars advocate conducting the left half colon resection to ensure blood supply for proximal colon.^8^ Good blood supply of the proximal colon is ensured after the tumor is cut off by low-position Dixon operation. Anastomotic leakage is unavoidable if blood supply is poor even if good suture techniques are used. Our experiences lie in that 1) after tumor resection, maintaining visible arteriopalmus at the proximal anastomosis end was necessary. If arteriopalmus or pulsatile bleeding was invisible, adequately freeing the flexura coli sinistra was feasible; 2) the proposed cut sigmoid mesocolon was firstly separated and cut in the abdominal cavity up to the colonic wall. Subsequently, pelvic partial operations were conducted. These operations included thoroughly anatomizing the pelvic floor, mutilating rectums, carefully examining the pelvic bleeding, and washing the pelvic cavity. After about 30-60 min, the blood supply of the proposed sigmoid colon to be maintained was examined. During this time, the intestinal wall color showed an obvious dividing line of the intestinal wall color.

Absence of tension was ensured in anastomotic stoma. Anastomotic stoma without tension is the basis to ensure a good blood supply to the anastomotic stoma. Tension is usually from sigmoid the mesocolon. If the anastomotic stoma has slight tension, ligating the proximal root and cutting off the sigmoid arteries or changing the end-end anastomosis of rectum with sigmoid colon into the end-side anastomosis of rectum with sigmoid colon is feasible. Therefore, relieving partial anastomotic tension is feasible based on the blood supply. The decision whether the inferior mesenteric artery shall be cut off at the root is based on the stage of the tumor and the pathological type. The inferior mesenteric artery was cut off at the root and flexura coli sinistra was adequately freed only if the inferior mesenteric artery had a swollen lymph node at the root or the anastomotic stoma had tension.^8^ The level of anastomosis was accurate and reliable at the anastomotic stoma.

The mesenteries at the rear of the rectal wall and at the two sides at the proposed anastomotic site were removed completely. The mesorectum within the range of 1 cm away from the anastomotic stoma was thoroughly removed. Therefore, reducing first the local recurrence is feasible. Second, the level anastomosis of anastomotic stoma is accurate, which prevents anastomotic fistula occurrence.

As regards prophylactic colostomy, some scholars think that performing preventive enterostomy is necessary, including ileostomy and transverse colostomy, to prevent anastomotic fistula after low-position anastomosis. More people supported to this point of view in recent years. However, whether preventive enterostomy can prevent anastomotic fistula occurrence remains controversial.^10^^,^^11^ In recent years, more studies have suggested that preventive enterostomy can obviously prevent the occurrence of anastomotic leakage after low anterior resection of rectal cancer.^3^^,^^12^^-^^14^ In this study, only one case received preventive transverse colostomy because preoperative radiotherapy was conducted and rectal wall edema was more severe during operation. The other cases did not receive preventive enterostomy. We believe in the feasibility of conducting preventive ileum or transverse colostomy for partial particular patients, such as those receiving preoperative pelvic radiation therapy, patients with severe hypoalbuminemia and uncontrollable diabetes mellitus, and patients administering glucocorticoid for a long time. Preventive enterostomy is unnecessary for patients only receiving TME or laparoscopic operation. After super-low anastomosis, the anal drainage tube can be placed into the re-constructed anal tube rectum.^15^ Intestinal mechanical preparation is done according to the obstruction situations before operation to ensure an empty post-operative early anastomotic stoma at the proximal side. Generally, we provided liquid diet in the afternoon before the operation and oral copragogue after supper. Carefully observing the cathartic effect was necessary. Oral copragogue was administered additionally if cleaning was not ideal, and conducting routine cleaning of enema was needed. Recently, other reports on surgical techniques are available for the prevention of anastomotic leakage.^16^^,^^17^

***Actively treating complicated*** ***systemic diseases: ***Various reasons are provided for the occurrence of anastomotic fistula of rectal cancer after low-position Dixon operation.^18^ In clinical work, focusing attention on individual factor is forbidden, and performing both works in the perioperative period and focusing on each detail of the operative procedure well are necessary. Therefore, the incidence of anastomotic fistula will be reduced to the minimum extent. Unfortunately this study is a retrospective analysis, the number of cases is less, and some data is not perfect. Randomized, controlled and prospective studies should be performed to confirm our findings.

## Authors Contributions:

**Ming Xu **conceived, designed the protocol, and prepared the final manuscript.

**Feng Gao** did manuscript writing.

All the authors were involved in clinical management of patients.

**Ming Xu **takes the responsibility and is accountable for all aspects of the work in ensuring that questions related to the accuracy or integrity of any part of the work are appropriately investigated and resolved.
